# Hemorrhagic giant retinal cyst: a rare complication of a retinal vasoproliferative tumor

**DOI:** 10.22336/rjo.2026.19

**Published:** 2026

**Authors:** Alejandro Verdu Reyes, Jose Ignacio Vela Segarra, Carlos Oribio-Quinto, Anna Hermosa Masdeu, Marta Caminal i Carames, Pilar Piquer Perez

**Affiliations:** 1 Department of Ophthalmology, Hospital de la Santa Creu i Sant Pau, Barcelona, Spain; 2 Institut Condal d’Oftalmologia, Barcelona, Spain; 3 Universitat Autònoma de Barcelona, Bellaterra, Spain; 4 Institut d´Investigació Biomèdica Sant Pau (IIB SANT PAU), Barcelona, Spain

**Keywords:** giant cyst, hemorrhagic cyst, retinal tumor, retinal vasoproliferative tumor, RVPT = retinal vasoproliferative tumor, RVPTs = retinal vasoproliferative tumors, VEGF = vascular endothelial growth factor, Anti-VEGF = anti-vascular endothelial growth factor, SD-OCT = spectral-domain optical coherence tomography, RPE = retinal pigment epithelium, PPC = pars plana cysts

## Abstract

**Objective:**

To present a rare case of a giant cyst with intralesional hemorrhage associated with a peripheral retinal vasoproliferative tumor (RVPT).

**Methods:**

A 57-year-old male with vision loss in the right eye had an epiretinal membrane. Surgery revealed a yellowish-red mass with a large cyst in the retina, which was diagnosed as a peripheral RVPT. The lesion was observed until intralesional hemorrhage and progressive growth developed.

**Results:**

Intravitreal anti-VEGF injection reduced intralesional bleeding, and external cryotherapy achieved regression and stability of both the tumor and the cyst.

**Discussion:**

The differential diagnoses for this entity primarily included other retinal tumors and pars plana cysts. We suspected that this rare presentation of RVPT associated with a giant cyst might be due to contraction of the glial tissue. Intralesional hemorrhage resulting from vascular penetration inside the cyst was resolved with intravitreal anti-VEGF treatment, resulting in intrinsic vascular regression.

**Conclusion:**

Giant haemorrhagic retinal cysts are a rare, previously undescribed complication of peripheral retinal vasoproliferative tumours, in which anti-VEGF therapy may reduce bleeding.

## Introduction

Retinal vasoproliferative tumors (RVPTs) are benign retinal lesions containing both glial and vascular components. Recent histopathologic and immunohistochemical findings indicate that most of its components are glial and astrocytic, with the vascular component residual. As a result, some authors refer to this lesion as a reactive retinal astrocytic tumor or reactive retinal ganglion cell [1,2].

Approximately 50% of RVPTs are located in the inferotemporal quadrant of the retina, arising from the peripheral retina, with some extending to the ora serrata. Classically, these tumors have been classified as primary (idiopathic) or secondary (associated with other ocular diseases) [3,4].

This tumor appears as a solid yellowish-pink mass in the peripheral retina. A network of telangiectatic vessels is observed on the surface of the RVPT. Clusters of small aneurysms in the telangiectatic plexus, embedded within the tumors’ deeper matrix, can also be observed. Unlike retinal capillary hemangiomas, feeding vessels are neither dilated nor tortuous [5,6].

Due to their location, these patients initially have no symptoms. Still, they may develop secondary complications, including epiretinal and subretinal membranes, cystoid macular edema, hyperpigmentation of the retinal pigment epithelium, or exudative retinal detachments [5,7]. As a result of its vascular component, RVPTs may be surrounded by intraretinal hemorrhages, intraretinal or subretinal exudation, or vitreous hemorrhages [3].

Herein, we present a rare case of a giant cyst with intralesional hemorrhage associated with a peripheral RVPT, including its treatment and subsequent resolution. To the best of our knowledge, no similar cases have been previously described in the literature.

## Methods

**Fig. 1:**
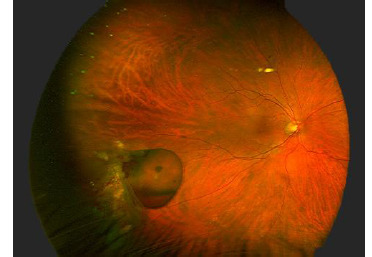
Fundus photograph showing a giant cyst with intralesional hemorrhage associated with a peripheral retinal vasoproliferative tumor

A 57-year-old male presented to our clinic with progressive vision loss and metamorphopsia that had developed over two months in his right eye. The best-corrected visual acuity was 20/50 in the right eye and 20/20 in the left eye. The anterior segment showed mild nuclear cataracts in both eyes. Fundoscopic examination of the right eye showed asteroid hyalosis in the vitreous cavity and an epiretinal membrane, confirmed by SD-OCT. Combined surgery, including phacoemulsification, intraocular lens implantation, and 25-gauge vitrectomy with peeling of an epiretinal membrane, was performed. During the vitrectomy, a yellowish-red mass with a large cyst on its surface was observed in the inferotemporal retina. This finding was consistent with that of peripheral RVPT (**Fig. 1**). Observation of the lesion was decided.

One year after the surgery, the patient presented to the emergency room with visual floaters in the right eye. Fundus examination revealed a mild vitreous hemorrhage with bleeding within the giant cyst. Blood occupied the inferior part of the cyst, with a hyphema-like aspect.

## Results

**Fig. 2:**
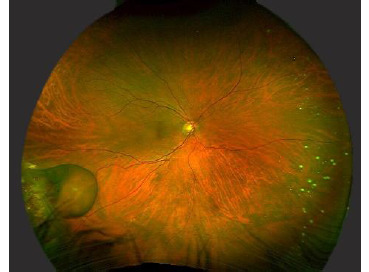
Disappearance of intracystic hemorrhage after bevacizumab intravitreal injection

The patient received an intravitreal injection of bevacizumab, and the intracystic hemorrhage resolved within 3 weeks (**Fig. 2**). Six months later, an increase in the size of the RVPT was observed, and external cryotherapy was performed. During follow-up, regression of both the tumor and the cystic lesion remained stable, and the best-corrected visual acuity was 20/30 in the right eye.

## Discussion

Numerous lesions can be incidentally found in the peripheral retina. Diagnosing these lesions can be challenging and relies primarily on ophthalmoscopic examination. It is essential to exclude conditions such as retinal capillary hemangioma, retinal and choroidal metastasis, amelanotic choroidal melanoma, and other exudative processes. Additionally, the presence of a large cyst associated with the peripheral lesion in our patient forced us to consider other cystic peripheral lesions, such as retinoschisis, retinal detachment, and pars plana cysts (PPC) as differential diagnoses [5,6,8].

Histological examination of the RVPTs has revealed metaplastic RPE cells along with abnormal glial tissue and blood vessels. These cells may transform into mesenchymal cells that produce collagen and modulatory factors that may contribute to tumor formation. These RPE cells may also have contributed to cyst formation in our case, and we hypothesize that the bleeding could be explained by contraction of the glial tissue [9].

Among the diagnoses we considered for the cystic lesion, PPC is relatively common, affecting up to 18% of the population. However, PPCs tend to be numerous and smaller posteriorly and fewer and larger anteriorly. In fact, larger cysts extend from the ciliary processes to the ora serrata, but the retina is not the origin of these lesions [8]. In our case, the large cyst clearly arose from the tumor rather than the pars plana or the ora serrata.

Another similar diagnosis to consider was retinoschisis, which typically has a smooth border, visible blood vessels, and is located within the inner retinal layers. These vessels were not observed in our patient. Instead, they penetrated the cyst through its supporting base. Thus, we believe that in our patient, the cystic lesion and the bleeding within it originated directly from the internal structure of the tumor.

Anti-VEGF treatment likely worked by reducing the tumor’s intrinsic vascularization [10]. However, given the cyst’s subsequent enlargement, a more definitive treatment was required to prevent further bleeding.

## Conclusion

In conclusion, giant cysts with intrinsic bleeding are rare complications that have not been described in the literature to date and can develop in association with peripheral RVPTs. In our case, intravitreal anti-VEGF injection reduced intralesional hemorrhage; however, due to progressive enlargement of the lesion, external cryotherapy was mandatory to achieve regression of both the tumor and cyst.
